# Genome Annotation Transfer Utility (GATU): rapid annotation of viral genomes using a closely related reference genome

**DOI:** 10.1186/1471-2164-7-150

**Published:** 2006-06-13

**Authors:** Vasily Tcherepanov, Angelika Ehlers, Chris Upton

**Affiliations:** 1 Department of Microbiology and Biochemistry, University of Victoria, Victoria, BC, V8W 3P6, Canada

## Abstract

**Background:**

Since DNA sequencing has become easier and cheaper, an increasing number of closely related viral genomes have been sequenced. However, many of these have been deposited in GenBank without annotations, severely limiting their value to researchers. While maintaining comprehensive genomic databases for a set of virus families at the Viral Bioinformatics Resource Center  and Viral Bioinformatics – Canada , we found that researchers were unnecessarily spending time annotating viral genomes that were close relatives of already annotated viruses. We have therefore designed and implemented a novel tool, Genome Annotation Transfer Utility (GATU), to transfer annotations from a previously annotated reference genome to a new target genome, thereby greatly reducing this laborious task.

**Results:**

GATU transfers annotations from a reference genome to a closely related target genome, while still giving the user final control over which annotations should be included. GATU also detects open reading frames present in the target but not the reference genome and provides the user with a variety of bioinformatics tools to quickly determine if these ORFs should also be included in the annotation. After this process is complete, GATU saves the newly annotated genome as a GenBank, EMBL or XML-format file. The software is coded in Java and runs on a variety of computer platforms. Its user-friendly Graphical User Interface is specifically designed for users trained in the biological sciences.

**Conclusion:**

GATU greatly simplifies the initial stages of genome annotation by using a closely related genome as a reference. It is not intended to be a gene prediction tool or a "complete" annotation system, but we have found that it significantly reduces the time required for annotation of genes and mature peptides as well as helping to standardize gene names between related organisms by transferring reference genome annotations to the target genome.

The program is freely available under the General Public License and can be accessed along with documentation and tutorial from .

## Background

With recent advances in DNA sequencing technology and reductions in sequencing costs, it has become relatively easy to sequence the complete genomes of many viruses and it is not uncommon for researchers to determine the sequence of multiple virus isolates as part of a single experiment. Although this ability to gather larger collections of genome sequences has opened up new avenues of research, it has also led to significant problems related to data management and sequence annotation. Examples of this data explosion include the following, all found in GenBank: 1) 1201 nearly complete genomes of human immunodeficiency virus (HIV); 2) 53 complete poxvirus genomes, with genomes ranging in size from 134 – 360 kb; 3) more than 125 SARS genomes submitted since the first two SARS coronavirus genomes were published in May, 2003.

The fact that the value of genomic data extends far beyond its use in an original publication is the foundation of *data mining *experiments. Unfortunately, however, a large number of the complete virus genomes submitted to GenBank lack annotations, severely limiting the usefulness of the data. The subsequent annotation of these genomes by multiple researchers, who may lack bioinformatics experience, is a tedious and time-consuming process. In order to facilitate the process of annotation, we have developed a tool, Genome Annotation Transfer Utility (GATU), which makes use of the fact that most unannotated genomes are closely related to previously annotated genomes. The application can be run on most major operating systems including Mac OS X, Windows and Linux.

Although a similar program, Sequin [1], can transfer annotations between two related sequences, these sequences must be co-linear and aligned. For example, Sequin can be used to transfer annotations between highly similar HIV genomes that have identical gene content. However, Sequin is not designed for use with larger viruses such as poxviruses and herpesviruses; the genomes of these viruses are far more variable, and contain many non-essential genes not conserved between closely related viruses. For such viruses, GATU is ideal, as it does not require the two input genomes to be aligned and can handle significant variations (e.g. sequence inversions) between the two genomes.

To summarize, GATU has been designed to fill a gap in the currently available software repertoire. By automatically transferring gene annotations that have very similar orthologues in closely related genomes, it reduces much of the tedious, time-consuming task of annotation while still leaving critical decisions in the hands of the researcher. Further, it does not require the target and reference genomes to be aligned and is able to deal with significant differences between the genomes. GATU does not, however, use sophisticated tools to make gene predictions because the simplicity of viruses, which have relatively small genomes, and availability of well annotated reference genomes make this unnecessary. Instead, GATU is intended to address the problem database curators face in attempting to annotate a large number of closely related genomes.

## Implementation

### Design rationale

Before implementation of GATU, the following goals were set for the program: 1) provide automated transfer of annotations, but do not take decision-making out of the hands of the annotator; 2) provide a simple platform-independent graphical user interface (GUI); 3) reuse tools familiar to researchers in the field in order to lessen the learning curve; 4) when feasible, pre-calculate alignments to reduce the waiting time during the hands-on checking of annotations.

GATU was implemented in Java to allow its use with multiple operating systems. Users simply launch the application (the client) from a web page using Java Web Start. This downloads a copy of the program to their system and opens the application, thereby avoiding most installation problems. If an updated version of the program has been released since it was last accessed, this version is automatically downloaded upon starting the program; this feature eliminates the need for users to check for updates. Java Web Start is included in the Mac OS X operating system and can be easily installed on other operating systems in a few minutes. Help and instructions are available on our website [2]. Furthermore, coding in Java allows interoperability of GATU with existing Java-based applications developed by our group, including Base-By-Base (BBB) [3] and Viral Genome Organizer (VGO) [4].

### Components

GATU consists of two distinct components. The first is a Java Swing-based GUI that allows the user to select the reference and target genomes, initiate automatic transfer of annotations, view and evaluate the results, and finally select the ORFs to be annotated. The second is an application server, which runs programs such as BLAST [5], NEEDLE [6,7], and CLUSTALW [8] on a remote server and then returns the results to the client machine. The user may choose not to use our application server and run BLAST, NEEDLE and CLUSTALW on the local machine if the appropriate programs are installed on that machine. The complete instructions on how to do that are available from our documentation page.

The GATU GUI consists of five sections within a main window: 1) a menu bar that provides access to *Help *texts, *Preference *settings, etc.; 2) a genome selector with which the user chooses the reference and target genomes; 3) the *Annotations area*, which shows the annotations of both genomes, BLAST and NEEDLE results, and ORF predictions; 4) the *Genome map *sub-window, which displays a graphical view of the target genome and/or the reference genome and their associated annotations; 5) and a set of *action buttons *to access the supporting applications (VGO and BBB), initiate annotation, and finally save the annotation results to GenBank, EMBL or XML files.

The role of the *Application Server *is to link the GATU client to the database search and sequence alignment applications the program uses; for convenience and enhanced speed, these may be installed on a more powerful machine or on a cluster. The process operates in the following simple manner. The client system sends a request to the *Application Server *to run a specific program (such as BLAST); the Application Server then runs the program using the client's input and subsequently passes the output of the program back to the client machine, where it is displayed to the user in the GUI. The primary goal of this client-server arrangement is to allow the client to run a wide variety of bioinformatics applications without the need to have those applications installed on the machine where the client resides. This optimizes speed, reduces the RAM required for the client machine, and reduces the problems associated with cross-platform support.

### Annotation

To initiate the process of transferring annotations using GATU, the user selects the reference genome (a file in GenBank format) and the target genome to be annotated (a file in FASTA format) and then clicks the *Annotate *button; the reference and target genomes reside on the local/client machine. The program will then begin the annotation routine. The first step is to use each ORF in the reference genome as a query to search the target genome. GATU runs the following searches: 1) TBLASTN for intron-less genes and mature peptides (i.e. a final peptide or protein product following post-translational cleavage) and 2) BLASTN with the exons of intron-containing genes. The alignments returned by the BLAST searches are used, together with a list of putative ORFs (longer than the specified threshold), to infer potential annotations for the target genome.

**Figure 1:**
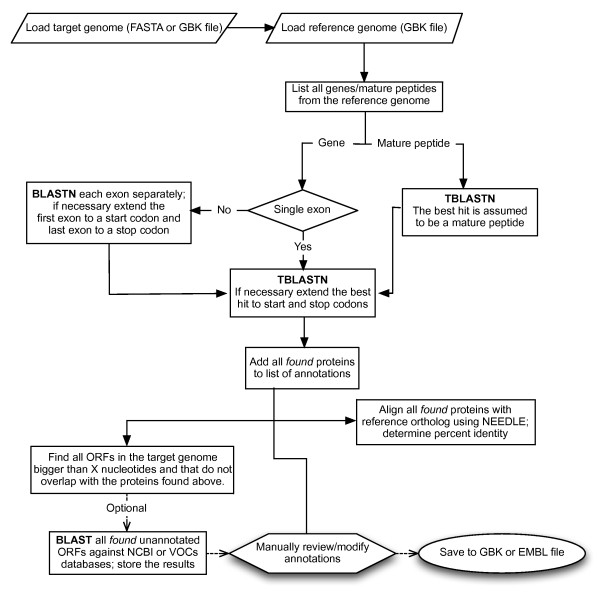
GATU process flow chart.

Of note, is that GATU allows the user to review the BLAST alignments together with the reference genes used in this process; for all the suggested annotations that are displayed in the *Annotation window*, links are provided to the search and alignment results. The overall annotation algorithm is shown in Figure 1. For each reference gene tested, GATU takes the start and stop positions of the best BLAST match in the target genome; if necessary, it then extends the ends of this exon in both directions in the same reading frame until the nucleotide sequence contains a start or a stop codon. Exons in the reference genome that lack either a start or a stop codon are excluded from this process, along with mature peptides. The region found in this manner is translated into a peptide sequence; NEEDLE then generates a global alignment between this translated sequence and the corresponding protein from the reference genome. If the similarity score for the alignment exceeds the cut-off value for acceptance (which can be changed by selecting *Preferences *from the menu), the "*Accept annotation" *box for this gene will be pre-checked to simplify the process for the user.

Since it is possible that the reference genome may have not been fully or correctly annotated, or that the target genome contains ORFs that are not present in the reference genome, GATU also finds all possible target genome ORFs. These are defined simply as any nucleotide sequence starting with ATG and ending with a STOP codon; all those that have not already been matched to a reference genome ORF are displayed as *Unassigned-ORFs*. The user may enter the minimum required length for these ORFs (default is set to 180 nt) and can also choose not to show *Unassigned-ORFs *that overlap significantly with regions that contain significant gene matches in the reference genome. BLAST searches of the *Unassigned-ORFs *against the NCBI database can be run automatically or manually, as required.

Once this automated process is complete, the user is then able to review the suggested annotations and apply any modifications deemed necessary. GATU allows the user to review the BLAST and NEEDLE alignments as well as the reference genes used; it simplifies this process by caching all of the previously performed searches and alignments; these can be instantly obtained by selecting the ORF and clicking the appropriate button.

In addition to transferring the location of the genes from the reference genome, GATU also takes the associated *Product name *from the GenBank file and displays it in the main annotation window; it can be edited if required, and is exported to the final annotation output file.

## Results and discussion

### Basic use of GATU

**Figure 2:**
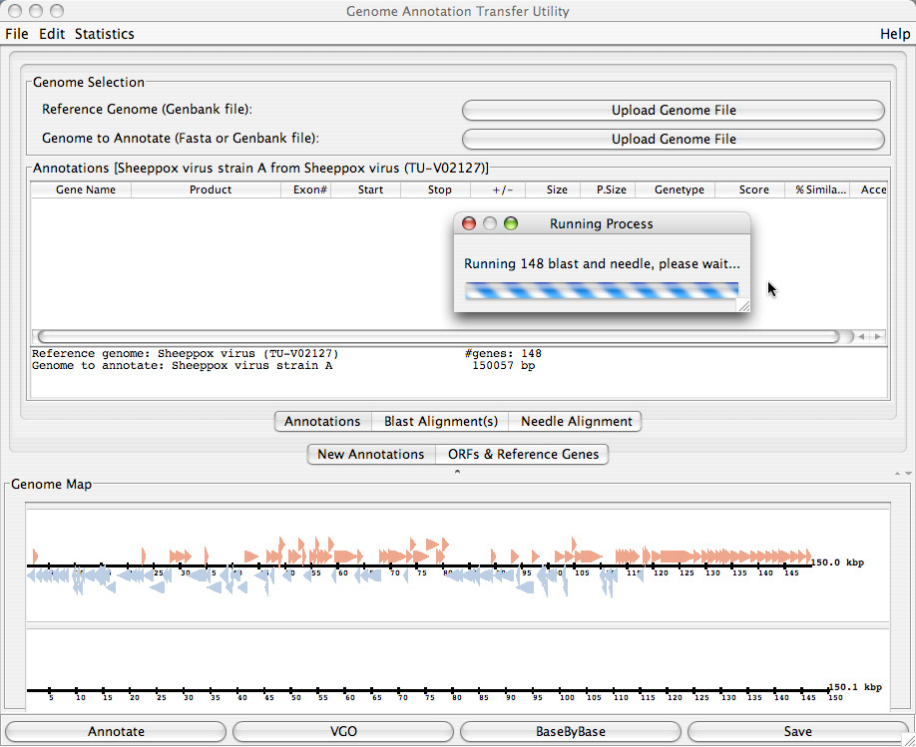
GATU GUI screen shot after loading genomes and clicking *Annotation *button; the annotations that have been read from the reference genome GenBank file are displayed.

The utility of GATU is illustrated here by the annotation of sheeppox virus strain A (SPPV-A) which was deposited to GenBank unannotated (AY077833). The related SPPV strain TU-V02127 (NC_004002) is used here as the reference genome. The annotations are extracted from the reference genome and displayed graphically (Figure 2). The SPPV genomes are approximately 150 kb in length; running on one processor of a dual 1.0 GHz Macintosh G4, the automated process took less than 15 min to complete, with no user intervention required during this time. However, if a BLAST search is also performed against the NCBI nr database for each ORF, the running time will be substantially longer; we routinely run these searches interactively since only a small fraction of the ORFs require them.

**Figure 3:**
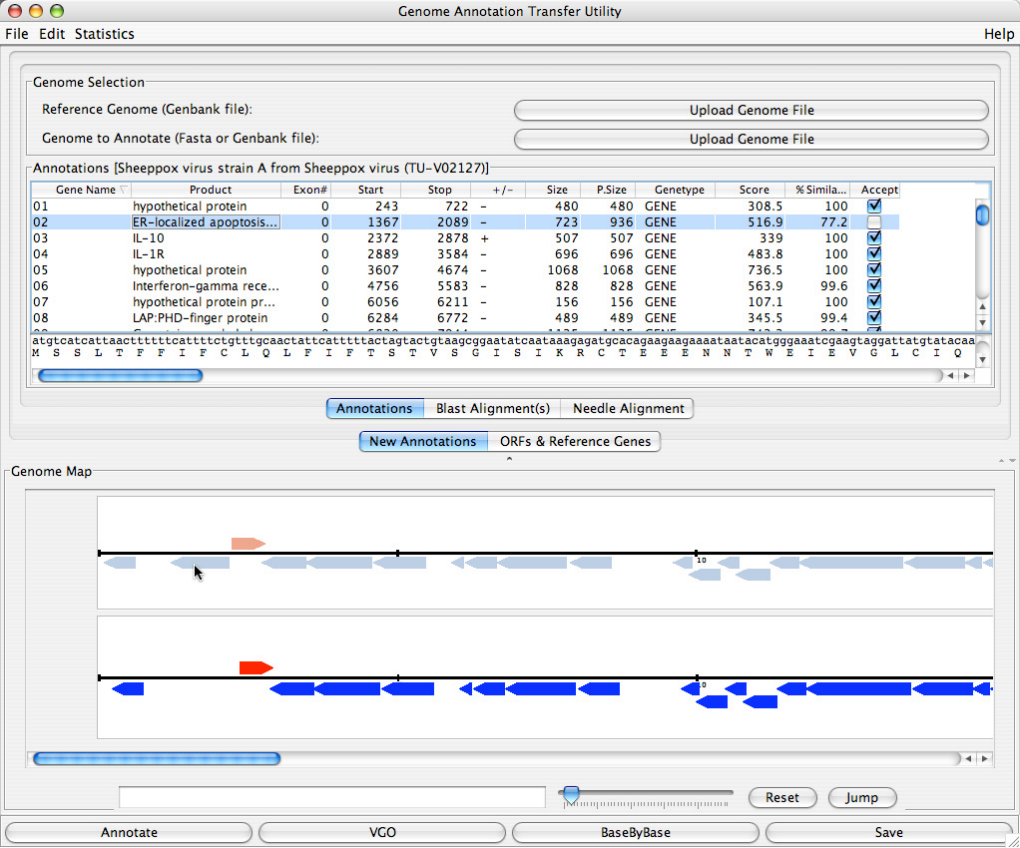
List of ORFs automatically annotated by GATU. One ORF that was not detected in the target genome is highlighted in blue in the top panel of the main GATU window; the *Accept *button is not selected. There is no corresponding ORF in the bottom half of the panel representing the target genome.

After processing is complete, GATU provides the user with an interactive table (Figure 3, top section) and a graphical view (Figure 3, bottom section) of annotations; the views in these panels can be modified in the *Preferences*. Clicking on a row in the table (containing an accepted annotation) will automatically highlight this annotation in the graphical view; the *Jump *button moves the graphical display to center the ORF in the window. The slider at the bottom of the graphical view allows the user to zoom in and out. The interactive table contains a list of all the putative annotations for the target genome, along with relevant information for each annotation; clicking on the column header will sort the table by the column value. The buttons below the interactive table allow the user to switch between lists of the *Reference genes *and target genome *Annotations*.

The interactive table and graphical display allow the user to review the automatically generated annotations and accept or reject them as desired. To aid the user, GATU pre-selects the *Accept annotation *box for all annotations that meet user-specified requirements of length, percent sequence identity and coding strand identity. In our example, GATU found and automatically accepted target sequence counterparts of 146 of the 148 genes (genes 1–147 and gene 101a) present in the reference genome. The accepted ORFs were 99.1–100% similar (predicted amino acid sequence) to the reference genes and 127 were 100% similar; this also indicates that the start/stop positions of the reference and target genes matched. Variation in the start/stop positions can also be examined by comparing the *P. size *column (predicted size) with the *Size *column (actual length in the reference genome) and viewing the NEEDLE alignments.

**Figure 4:**
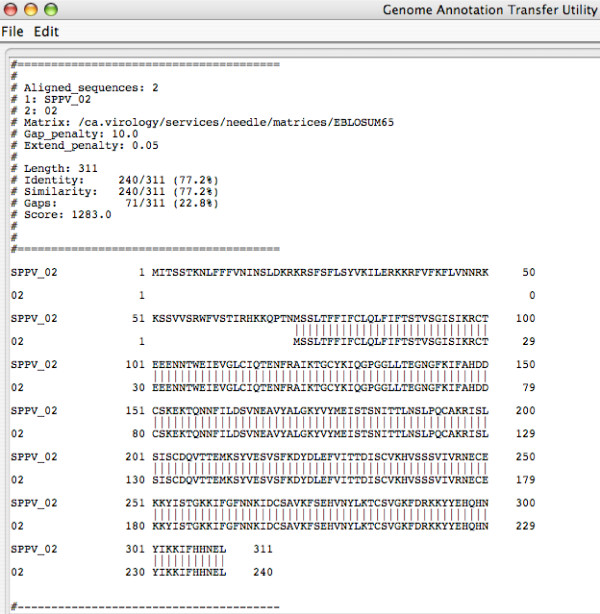
Results of a NEEDLE alignment run with an *Unassigned-ORF*; display is presented in the main GATU window.

**Figure 5:**
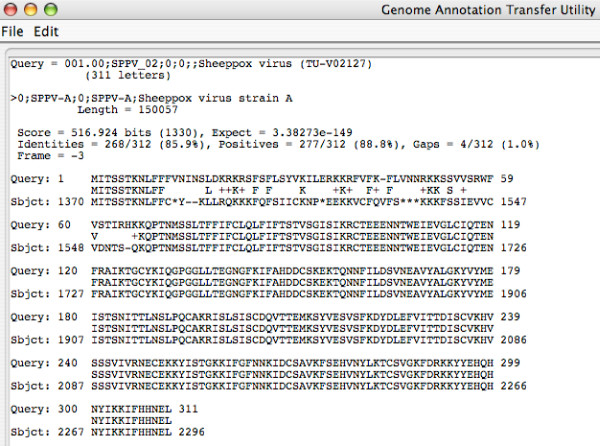
Results of TBLASTN search with an *Unassigned-ORF*; display is presented in the main GATU window.

Before deciding which annotations to include in the genome file, the user may wish for more information about a particular annotation. In our example, genes 02 and 146 (these two genes happen to be identical because they are present in the terminal inverted repeats of the virus) need reviewing, as they were not automatically accepted for inclusion; the user will have to determine whether they should be accepted. To assist with this task, a global alignment of the reference protein and its putative counterpart on the target genome (generated by the NEEDLE program) can be obtained by clicking on the *Needle Alignment *button (Figure 4). The NEEDLE alignment shows that the ORF in the target genome is truncated at the N-terminus but contains the remaining 240 aa encoded by the reference genome. This global alignment also provides a useful indication as to the level of similarity between the two ORFs. Another useful tool is a TBLASTN search of the target genome using the reference gene as a query; the results of this search can be obtained by clicking on the *Blast Alignment(s) *button (Figure 5). From the data shown, it is apparent that a frame-shifting mutation is responsible for the difference between the target and reference ORFs. If desired, the user could open these two genomes in our Viral Genome Organizer (VGO) program to determine if the promoter regions are similar and the position of the frame-shifting mutation (located at a run of Ts). Another application that users will find useful at this stage is JDotter, which can show an alignment of the genomes together with the whole genome dotplots [9]. With these data at hand, the user can now make an informed decision as to whether this putative ORF should be included in the target genome annotations. Given that the promoter regions and ORF start sites are similar, it is likely that protein translation of this mRNA would begin at the same position as in the reference genome, leading to the synthesis of a polypeptide only 12 aa in length. Therefore, this ORF should be omitted from the annotation even though, at first glance, it appears to be significant. Alternatively, the annotation could be accepted with the *FRAG *(fragment) designation; the user can select this by clicking in the relevant row of the *Genetype *column.

**Figure 6:**
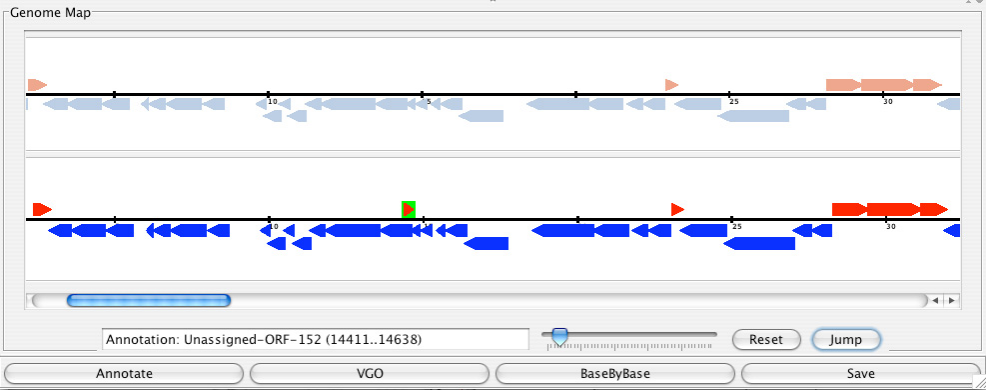
Genome map panel of GATU interface. Display of an *Unassigned-ORF *after temporarily selecting *Accept *box and clicking the *Jump *button; ORF is shown with green highlighting.

To complement the process of matching known genes in the reference genome to the target, it is necessary to search for potential ORFs in the target genome that have no obvious match in the reference genome. Such ORFs could be the result of additional sequences in the target genome, minor sequence differences in the reference genome resulting in the loss of a functional gene, errors in the original annotation, overlapping ORFs, or failure to use the first MET codon as the ORF start. All ORFs larger than the *Cutoff size *that have not been matched to a gene in the reference genome are automatically placed in an *Unassigned-ORFs *table, which is accessed by clicking on the relevant button below the interactive table. *Unassigned-ORFs *can be added to the list of selected annotations and the graphical display by checking the *Accept *box for a given ORF; the *Jump *button moves the graphical display to show the selected ORF in the window (Figure 6). Several utilities have been built into GATU allowing the user to further investigate the nature of these *Unassigned-ORFs*. Right-clicking on any of the *Unassigned-ORFs *and selecting the appropriate option allows the user to view the results of pre-run BLAST searches for the ORF, run BLAST searches if the *Manual BLAST search *option was checked during the initial annotation run, or initiate a new search using a different algorithm (e.g. TBLASTN) or database (e.g. *VOCs virus databases *[2], or the NCBI nr database).

In summary, GATU correctly transferred and automatically accepted annotation for 146 of 148 genes from the reference sheeppox genome to the target genome. The missing genes (2 copies of a gene in the terminal inverted repeats of the viral genome) encode a protein of only 36 aa and were subsequently detected by a TBLASTN search of the target genome. In further tests, 97% of the genes in rabbitpox virus (AY484669; a strain vaccinia virus) were correctly annotated by using vaccinia virus strain WR (NC_006998) as the reference genome, whereas 88% of the genes in rabbitpox virus were correctly annotated by using ectromelia virus (a different species in the same *Orthopoxvirus *genus) as reference (data not shown). It should be noted, however, that although duplicated genes, such as those in poxvirus terminal inverted repeats, are detected by GATU, the evaluation of paralogues requires special attention because GATU can only match a reference gene to the first BLAST hit that is found. GATU places other paralogues into the *Unassigned*-ORFs group where they can be reviewed by the annotator and manually added to the annotation.

### Annotation of bacterial genomes with GATU

Since many problems associated with genome annotation are magnified considerably when dealing with bacterial genomes due to their large size, we have tested GATU on bacterial genomes up to ten times the size of a typical poxvirus genome. First, *Chlamydia pneumoniae *strain TW183 (NC_005043) was annotated using *C. pneumoniae *strain AR39 (NC_002179) as the reference genome, containing 1112 annotated genes in its GenBank file. These two strains are highly similar (>90% nucleotide identity). Of the 1112 reference genes, 817 were 100% similar (global alignment with NEEDLE) to a gene found on the target genome, 145 genes were 95–99% similar, 28 genes were 90–94% similar, 22 genes were 85–89% similar, 16 genes were 80–84% similar, 11 genes were 75–79% similar, 14 genes were 70–74% similar and 59 genes were <70% similar. This information is available from the *Statistics *menu within GATU. With a minimum threshold of 60% aa similarity between the reference gene and the target ortholog, 1063 ORFs (96%) were accepted automatically by GATU and a further 24 were accepted from the reference genome after reviewing data in the *Unassigned ORF *table. Although annotation of bacterial genomes is outside of our area of expertise, another 29 ORFs that were not annotated in the reference genome were added from the *Unassigned-ORF *table to the target genome based on their similarity to genes in the NCBI nr database. In total, we were able to annotate the *C. pneumoniae *TW183 genome with 1116 genes and gene fragments; in comparison, the GenBank file for this genome contains 1113 annotations (NC_005043). Additional file 1 [Additional file 1] contains two Excel spreadsheets showing a more detailed comparison of these annotations; in summary, our annotation performed with GATU added several annotations to the target that were not in its GenBank file and failed to predict a small number of short ORFs. However, such differences will arise whenever two authors annotate a genome using different processes and standards; the discrepancies seen here represent less than 10% of the total genes, indicating a high level of consistency between the two annotations.

For our second test using bacterial genomes, we used two different species from the *Thermoplasma *genus; *T. volcanium *strain GSS1 (NC_002689) was annotated using *T. acidophilum *strain DSM 1728 (NC_002578) as a reference. The reference genome GenBank file contained 1482 annotated genes, and based on a threshold of 60% aa similarity 1103annotations (74%) were accepted automatically into the target genome annotation (11 genes had 100% similarity, 135 genes were 95–100% similar, 182 genes were 90–94% similar, 164 genes were 85–89% similar, 186 genes were 80–84% similar, 141 genes were 75–79% similar. 120 genes were 70–74% similar and 459 genes were <70% similar. A further 124 ORFs could be added from the *Unassigned-ORFs *table; most of these ORFs were truncated and/or fragmented versions of their counterparts in the reference genome. Finally, a number of target genome-specific ORFs (201) were added following further analysis of the *Unassigned-ORFs*, to yield a total of 1428 ORFs accepted for annotation. A comparison between our annotation of GSS1 using GATU and the annotated GSS1 GenBank file (NC_002689) showed 100 differences, approximately 7% [see Additional file 2].

The 1112 and 1482 BLAST searches/NEEDLE alignments required for these two annotation trials took 25 and 40 minutes, respectively, to run on a 1 GHz G4 Macintosh computer. In both cases, a large fraction of the total bacterial ORFs present were automatically annotated correctly by GATU; the majority of the "missed" ORFs were small (<150 nt) hypothetical genes that were either not annotated in the reference genome or were unique to the target genome. This represents a substantial reduction of the time and effort required for annotation, allowing the annotator to concentrate on those areas of the genome that require expert knowledge to correctly annotate.

## Conclusion

Although GATU is not a comprehensive genome annotation system such as Pedant [10] or Manatee [11], GATU significantly reduces the annotation workload by automatically transferring over 90% (depending on the similarity of the reference and target genomes) of the annotations from a reference genome to the target. GATU was designed for use with viral genomes, but as demonstrated here, GATU is also useful for annotation of bacterial genomes. Furthermore, GATU offers a variety of built-in tools to assist the user in assigning novel annotations.

Currently, the client-server nature of GATU relies on the use of our server for searches; high-throughput users can contact the authors for local installation instructions. In addition, we have also modified GATU to run as a simple stand-alone application that does not connect to the viral databases at the VBRC [2].

## Availability and requirements

**Project Name: **GATU

**Project Home Page: **GATU may be accessed from the workbench at 

**Operating Systems: **All platforms supporting Sun's JRE version 1.4.1 or compatible

**Programming Languages: **Java, SQL

**Other requirements: **Java 1.4 or higher

**License: **GNU General Public License

**Restrictions For Non-Academic Use: **Contact corresponding author

**Tutorials: **A flash based tutorial is available from the  website

## Authors' contributions

VT and CU described and specified the features of, and problems to be solved by GATU, tested the program, and provided usage examples; AE implemented coding of the software; all authors contributed to writing of the manuscript.

## Supplementary Material

Additional File 1 **Additional file 1 **is a Microsoft Excel spreadsheet containing *Chlamydia pneumoniae *strain TW183 open reading frames transferred from *C. pneumoniae *strain AR39 (NC_002179) and accepted for annotation by GATU. The annotations are compared to genes of *C. pneumoniae *TW183 found in the GenBank file (NC_005043). Click here for file

Additional File 2 **Additional file 2 **is a Microsoft Excel spreadsheet containing *T. volcanium strain *GSS1 open reading frames transferred from *T. acidophilum *strain DSM 1728 (NC_002578) and accepted for annotation by GATU. The annotations are compared to genes of *T. volcanium *GSS1 found in GenBank file (NC_002689). Click here for file
